# Effect of Preconception Care Intervention on Maternal Nutritional Status and Birth Outcome in a Low-Resource Setting: Proposal for a Nonrandomized Controlled Trial

**DOI:** 10.2196/28148

**Published:** 2021-08-16

**Authors:** Dharitri Swain, Jasmina Begum, Swayam Prangnan Parida

**Affiliations:** 1 College of Nursing All India Institute of Medical Sciences Bhubaneswar India; 2 Department of Obstetrics & Gynecology All India Institute of Medical Sciences Bhubaneswar India; 3 Department of Community Medicine and Family Medicine All India Institute of Medical Sciences Bhubaneswar India

**Keywords:** preconception care, maternal nutritional status, birth outcome, paternal preconception health, childbirth, birth outcomes, maternal and child health, maternal health, maternal and child nutrition, health education, pediatrics

## Abstract

**Background:**

The provision of preconception care approaches such as maternal assessments and education on healthy lifestyle (including physical activity, nutrition, and dietary supplements such as folic acid), general and sexual health, avoidance of high-risk behavior, and immunizations has been shown to identify and reduce the risk of adverse birth outcomes through appropriate management and preventive measures.

**Objective:**

The goal of the study is to determine the effect of an integrated preconception care intervention on delivery outcomes, which is a novel challenge for lowering unfavorable birth outcomes in India’s low-resource setting. The main objectives are to investigate the relationship of birth outcomes to both maternal and paternal preconception health and determine the effect of preconception care intervention on improvement of maternal nutritional status and reduction of the risk of adverse birth outcomes such as prematurity, low birth weight, and maternal and neonatal complications.

**Methods:**

A nonrandomized controlled trial design will be used for comparing 2 groups: preconception care with a standard maternal health care (MHC) program and an integrated MHC program (without preconception care). Two rural field areas of Khordha district, Odisha, will be selected for conducting the study. The study will enroll 782 married women between the ages of 18 and 35 years with their spouses, with 391 women in each group. The couples will receive preconception care based on their health circumstances, and they will be followed up at 3-month intervals before pregnancy. Following pregnancy, they will be followed up for 8 prenatal monitoring and care visits as well as 6 weeks after delivery as part of the standard MCH program. The preconception care intervention package includes couples counseling, contraceptive education and distribution, sex education, lifestyle modification, and nutritional supplementation of iron and folic acid, along with multivitamins if needed.

**Results:**

The proposal was approved by the institutional ethical committee for conducting the study in June 2020 (Ref No: T/EMF/Nursing/20/6). Participants were enrolled in phase 1 in April 2021, phase 2 of offering preconception services will begin in August 2021, and study outcomes will be measured from 2023 to 2024.

**Conclusions:**

Through preconception care and counseling, the eligible couples will recognize, embrace, and implement the actions to improve their preconception health. Finally, it is expected that maternal and paternal health will have a significant impact on enhancing maternal nutritional status and birth outcomes.

**Trial Registration:**

Clinical Trials Registry–India CTRI/2021/04/032836; http://ctri.nic.in/Clinicaltrials/pmaindet2.php?trialid=48239&EncHid=&userName=CTRI/2021/04/032836

**International Registered Report Identifier (IRRID):**

PRR1-10.2196/28148

## Introduction

Maternal health care (MHC) is a cost-effective and clinically helpful method of preventing unfavorable birth outcomes. However, adverse birth outcomes remain a significant public health concern around the world, contributing to substantial morbidity, mortality, and increased health care costs [1]. The majority of this adverse effect happens in low- and middle-income countries (LMICs), with mortality rates being higher in rural and low-resource populations. Starting prenatal treatment late in the first trimester may make it more difficult to check for risk factors and prevent a negative birth result. The importance of maternal health prior to pregnancy (preconception care) is becoming more well acknowledged, and improving a woman’s health and preparation prior to conception may prevent or reduce the risk of adverse birth outcomes.

Preconception care for all women, as well as women with particular risk factors such as maternal obesity, diabetes, hypertension, depression, substance misuse, and occupational variables, has been shown to improve maternal and newborn health in the long run [2-4]. On the other hand, much less is known regarding fathers’ preconception influences on delivery outcomes. Paternal health variables, such as obesity, cardiovascular health, and job circumstances [5], have been linked to birth outcomes, notably birth weight [6-9] in some studies. More research is needed to understand how paternal health affects birth outcomes, as well as whether this process occurs independently or in tandem with maternal health [10].

According to the US Centers for Disease Control and Prevention [1] and American College of Obstetrics and Gynecology guidelines [11], preconception care intervention included maternal assessment, screening, supplementation with folic acid and iron, vaccination, lifestyle modification, and counseling. Improved pregnancy and delivery outcomes, such as fewer low birth weight or preterm infants, congenital abnormalities, and intrauterine growth restriction, are all examples of good preconception health [1,12,13]. The Centers for Disease Control and Prevention also recommends that preconception care be improved and consumer-focused research be conducted to promote preconception health and reproductive knowledge. The implementation of comprehensive preconception care in low-resource areas in the Indian situation has not been researched on a wide scale. More study is needed to discover best practices and the most efficient ways to administer integrated preconception care components in remote areas. Our research will be conducted in the rural communities of Odisha, India. One of the most significant challenges facing Odisha’s health system is reducing maternal and infant fatalities. With many efforts from the state under the Reproductive, Maternal, Newborn Child, and Adolescent Health campaign, the state maternal mortality ratio and infant mortality rate have decreased over time, according to the sample registration system reports from 2015 to 2017. The present rate of decline, however, is insufficient to meet the 12 5-year plan goals. According to India’s National Family Health Survey–4 (2015-16), the prevalence of low birth weight infants is high in tribal-dominated states, with Odisha reporting the highest number of low birth weight newborns compared to the national average [14]. Inadequate antenatal care services, a low number of antenatal visits, and poor health-seeking behavior, such as delaying timely intervention and accessing emergency obstetric care, were recently linked to the occurrence and prevalence of obstetric complications like preterm deliveries, prolonged labor, and low birth weight babies, according to a population-based study conducted in Khordha district of Odisha [15].

Despite the fact that there are 8 or more scheduled visits of standard prenatal care without preconception care, which is considered insufficient, 99% of maternal and neonatal death occurs in LMICs like India, with the majority of deaths occurring in rural and low-resource communities. As a result, an integrated MHC program has been proposed, which includes a specific plan throughout the preconception and prenatal periods, in order to determine its impact on improving maternal health and reducing adverse birth outcomes such as prematurity, low birth weight, and maternal and neonatal complications as well as to investigate the relationship between birth outcomes and both maternal and paternal preconception health.

## Methods

### Research Design and Study Setting

A nonrandomized controlled trial design will be used to assess the impact of a preconception care intervention plan for maternal nutritional status and birth outcome among married women aged 18 to 35 years. The research will be conducted in the Khordha district of Odisha, which is located in India’s eastern rural community. Khordha district has a population of 22.52 lakh (2.25 million) people, accounting for 5% of the total population of Odisha. The district’s rural population accounts for 52% of the total population, with females accounting for 48% and males accounting for 52% [16].

### Study Participants

Married pregnant women aged 18 to 35 years, gravida and parity of less than 5 and who will attend a minimum of 8 scheduled visits of prenatal monitoring will be recruited into the standard MHC group. The integrated MCH program will enroll married nonpregnant women aged 18 to 35 years with their partners, gravida and parity of less than 5, who intend to have a child within 1 year and will attend at least 3 preconception appointments and get preconception care at least once every 3 months.

Participants who will not able to attend the scheduled preconception visits and antenatal visits will be excluded from the study. The sample size for the study will be 652 couples, which was calculated by using sample size calculation software (Epi Info, CDC) for sample size estimation of nonrandomized controlled trials with 95% confidence level and 80% power and risk/prevalence ratio (0.42) of low birth weight baby as an adverse birth outcome associated with preconception care in a previous study [17]. We expect 20% to be lost to follow-up; therefore, the total required sample size is rounded to 782, and each group will be enrolled with 391 women with their partners.

### Recruitment Process

A nonrandomized cluster sampling will be used to select the population samples from the targeted population. Each rural community health center will be considered as a cluster and will be listed in the sampling frame. In the first stage, 2 clusters will be selected randomly from a sampling frame of all rural health centers, Khordha district, Odisha, and all eligible participants fulfilling the sampling criteria in those clusters will be listed in the sampling frame. One cluster will be exposed to the integrated MHC program (ie, women with their partners who will receive preconception care and prenatal care), and another cluster will receive the standard MHC program (ie, women who received prenatal care without preconception care). In the next stage, eligible couples will be selected in each cluster by a convenience sampling technique proportionate to the sample size. After obtaining consent for enrollment, the selected eligible couples will be interviewed, and preconception health will be assessed by a team of research groups consists of a research coordinator, field data collectors, nurse-midwife, and doctor. The preconception service will be given to the eligible couples and they will be followed up at 3-month intervals before pregnancy and then up to 8 scheduled visits of prenatal monitoring and delivery as provided under the standard maternal health care program.

### Measures

In the initial phase, data on preconception sociodemographics, health conditions, and health behaviors of the participants and their partners will be measured and will be followed until their delivery. In the next phase, the characteristics of the pregnancy and birth outcomes will be assessed.

- Sociodemographic characteristics: parent ages, educational level, socioeconomic status, previous pregnancy, and birth characteristics
- Preconception health conditions: 4 variables are included: BMI, diabetes, high blood pressure, depression. Screening of cases for identifying diabetes and high blood pressure and also diagnosed cases will be classified as having diabetes and high blood pressure. Depression will be measured using a depression scale. BMI will be calculated by measuring height and weight and categorized according to standard categories of normal weight, underweight, overweight, or obese
- Preconception health behaviors: maternal and paternal health behaviors such as substance use, fast food consumption, and physical activity are included in the study, which will be assessed through a structured self-reported format. Substance abuse will be measured in the form of the frequency of taking alcohol or drugs or smoking. Consumption of fast food will be measured by respondents’ reports of the number of days per week in which they typically eat fast food. Physical activity will be measured by responses to a series of items which ask if the participant is engaged in a variety of activities, such as bicycling, doing aerobics, playing team sports, participating in individual sports, walking, or any physical work
- Pregnancy characteristics and birth outcomes: pregnancy characteristics, nutritional status of the mother, and birth outcome are the outcome variables. The nutritional status of the mother will be measured in the form of BMI and hemoglobin level anemia. The birth outcome will be measured using the gestational age of the baby, birth weight, maternal and neonatal complications, and mode of delivery (normal vaginal delivery and cesarean delivery)

### Ethical Consideration

The proposal for conducting the study has been approved by the institutional ethical committee (Ref No: T/EMF/Nursing/20/6). Detailed information about their preconception health assessment and testing will be given to the study participants, and written consent will be obtained from them before proceeding to data collection. No such risks are involved in delivering the routine preconception advice and care related to pregnancy, and it will be given under the guidance of an obstetrician and nurse-midwife. Most women will be counseled and educated about their planned pregnancy and preparing for a better outcome. This trial is registered at the Clinical Trials Registry–India [CTRI/2021/04/032836].

### Project Implementation Plan

**Figure 1:**
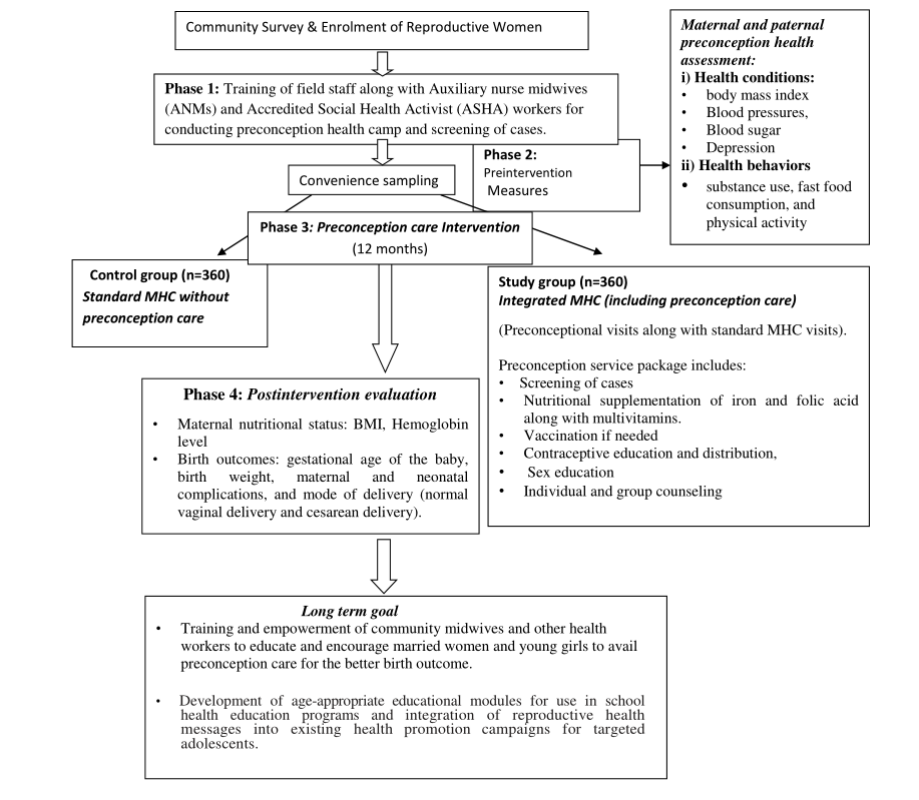
Phase-wise study work plan. ANMs: auxiliary nurse midwives; MHC: maternal health care.

Figure 1 depicts the phase-wise research implementation plan.

In the first phase, this project will collaborate with selected rural health centers for the implementation of effective preconception care intervention. Due permission will be obtained from the chief district medical officer, Khordha district, Odisha, and concerned community health workers such as accredited social health activists. Auxiliary nurse midwives will be involved in identifying eligible participants in their locality and bringing those couples for preconception advice and care at a free preconception health camp. Qualified data collection teams will be assigned to do the data collection in the targeted areas. The selection of the interviewers will be based on set criteria such as having a medical background or medical knowledge, familiarity with the region, fluency in the local language, and familiarity with surveys and data collection. All data collectors and field supervisors will be trained on questionnaires/assessment tools, processes of data collection, and ethical issues of the survey based on an already developed training plan. Also, community midwives will be trained and certified as trainees for trainers so that they will continuously involve the same type of preconception health assessment in their areas.

In the second phase, a preconception health camp will be conducted at their locality on a weekly basis until reaching the sample size to collect baseline data on preconception sociodemographics, screening of health conditions, and health behaviors of the enrolled participants. Community field testing for risk assessment will be done by the project team by involving community health workers.

In the third phase, preconception care service will be provided to the couples based on their health conditions and they will be followed up at 3-month intervals before conception. After conception, they will be followed up for 8 scheduled visits of prenatal monitoring and care and 6 weeks after delivery as provided under the standard MCH program. The detailed preconception care intervention plan is presented in Table 1 and includes couples counseling, contraceptive education and distribution, sex education, lifestyle modification, and nutritional supplementation of iron and folic acid along with multivitamins if needed. The preconception care training manual and its digital app will be available as technological support for women who prefer a self-directed approach for maintaining good preconception health for the better birth outcome.

In the fourth phase, a postintervention assessment will be done for measuring outcome variables such as the nutritional status of the mother during pregnancy and birth outcome. Maternal nutritional status includes BMI and hemoglobin level, and birth outcome includes gestational age of the baby, birth weight, maternal and neonatal complications, and mode of delivery (normal vaginal delivery and cesarean delivery).

**Table 1:** Preconception care intervention plan.

Service	Screening activities	Plan of action
Reproductive history and contraception	Inquire about previous pregnancies: Preterm birth, preeclampsia Congenital anomalies, stillbirth/miscarriage Gestational diabetes Caesarean birth, uterine anomalies, high/low birth weight Inquire about contraception: Interventions to delay age at first pregnancy and interpregnancy intervals	Provide appropriate referrals. Discuss family planning and conception. Advise women with prior cesarean delivery to wait at least 18 months prior to conception. Recommend folic acid 5 mg daily prior to conception and for 12 weeks after conception if positive history of neural tube defects. Recommend >12 and <60 month interpregnancy interval.
Sexual health	Sexually transmitted diseases	Provide treatment according to sexually transmitted infection guidelines. Inform women with genital herpes of the risk of vertical transmission.
Chronic medical conditions	Screen for diabetes, high blood pressure	Manage as per medical protocol
Mental health	Screen for the following conditions: Depression Anxiety Family history of mental health issues	Counsel women with mental health diagnoses of risks of pregnancy and relapse. Strategize management. Stabilize/optimize mood and anxiety level. Discuss risks and benefits of medications.
Medications	Screen for teratogenic medication use: Prescribed medications Over-the-counter medications Complementary and alternative therapy (herbal, natural, weight loss, athletic products or supplements, etc)	Potentially teratogenic medications should be changed to safer options. Women should be counseled not to stop prescribed medications without consulting with their provider. Recommend folic acid 5 mg daily prior to conception and for 12 weeks after conception for women taking folate antagonists (eg, methotrexate, sulfonamides, and antiepileptic).
Nutrition	Screen for issues regarding access to food, nutrition, storage, cooking facilities, and folic acid. Screen for iron-deficiency anemia if at risk.	Recommend folic acid 0.4-1.0 mg daily (through a multivitamin or supplement) and a folate-rich diet prior to conception and throughout pregnancy. Recommend calcium 1000 mg daily through food and/or supplements. Recommend an essential fatty acid–rich diet, including omega 3 and 6. Recommend avoiding raw/undercooked meat and fish and unpasteurized milk and cheese. Limit caffeine to <300 mg/day. Recommend vitamin D 600 IU (15 μg) supplementation daily. Recommend 2.6 μg of vitamin B12 daily through supplement or multivitamin. Provide referral to a dietitian or appropriate community agencies for nutritional support.
Vaccinations	Rubella, hepatitis B, varicella	Provide all immunizations required prior to conception with the exception of the flu vaccine, which can be administered before and/or during pregnancy.
Family and genetic history	Family history of a genetic condition such as consanguinity (first cousins or closer) or children who died at a young age (may reveal a metabolic condition) History of sudden unexplained death (may indicate cardiomyopathy or metabolic condition) History of infertility, multiple miscarriages (>3) Congenital malformations, birth defects Developmental delays, learning disabilities	Recommend folic acid 5 mg daily prior to conception and for 12 weeks after conception if positive family history of neural tube defects or high-risk ethnic group. Provide referral to a specialist for those with family and genetic history risk factors.
Weight status	Screen BMI (kg/m^2^) annually.	Underweight (BMI <18.5) Overweight (BMI 25-29.9) Obese (BMI >30) Recommend folic acid 5 mg daily prior to conception and for 12 weeks after conception for obese individuals. Discuss recommended healthy weight gain diet plan as per the BMI during pregnancy. Provide appropriate referrals for management.
Physical activity	Assess series of items in which the participant engages in a variety of activities, such as walking, doing other physical work, etc.	Recommend at least 150 minutes of moderate to vigorous aerobic physical activity per week in episodes of 10 minutes or more. Add muscle and bone-strengthening activities at least 2 days per week.
Substance use	Screen for tobacco (all forms), tobacco exposure (second-hand smoke), alcohol, other substances.	Counsel women with tobacco addictions of risks to pregnancy. Strategize management as required. Recommend an extra 35 mg of vitamin C daily for smokers. Provide brief intervention and appropriate referrals. Inform women of available patient resources.

### Data Analysis Plan

The data will be cleaned, validated, and analyzed using SPSS (version 20, IBM Corp). Descriptive statistics for continuous variables (mean and standard deviation) or categorical variables (frequencies) will be presented for participant characteristics and the outcome measures. Regression analysis will be used to examine potential associations between maternal and paternal preconception health and birth outcomes. Inferential statistics will be used for testing the effectiveness of preconception care intervention on maternal nutritional status and birth outcome.

### Expected Outcomes

There will be a strong impact of maternal and paternal health on birth outcomes such as gestational age of the baby, birth weight, maternal and neonatal complications, and mode of delivery (normal vaginal delivery and cesarean delivery). The preconception care intervention will improve birth outcome and nutritional status of the mother. The eligible couples will recognize, accept, and include the measures to improve their preconception health through preconception counseling and health teaching. Ultimately, it is anticipated that community midwives will be trained for disseminating effective preconception care in low-resource setting communities, which may bring better birth outcomes.

## Results

The proposal was approved by the institutional ethical committee for conducting the study in June 2020. Enrollment of participants to phase 1 began in April 2021, phase 2 of providing preconception service will begin in August 2021, and study outcomes will be measured from 2023 to 2024.

## Discussion

### Summary

Preconception health is associated with infant birth outcomes, which in turn influences health status throughout the lifetime. Some of the prepregnancy health conditions such as underweight, history of chronic hypertension, poor prepregnancy physical function, and smoking before pregnancy increase the risk of preterm birth and prematurity [18]. Maternal and paternal diabetes status demonstrated some of the strongest relationships with infant birth weight and gestational age. Interestingly, maternal diabetes was associated with increased birth weight, but paternal diabetes was associated with decreased birth weight [9,19-21]. Nationally, diabetes is becoming more common among young adults [18]; accordingly, diabetes management will become even more important for preconception care. The presence of elevated blood pressure in the mother was linked to a higher child birth weight. High blood pressure before conception [19,22] and during pregnancy [23,24] has been linked to a lower birth weight in previous research. Our research will also look into how preconception health issues like BMI, diabetes, and blood pressure affect delivery outcomes.

A prospective longitudinal study was conducted to see how maternal and paternal preconception health factors and behaviors affect infant birth weight and gestational age. Infant gestational age was found to be marginally lower for infants born to mothers with greater levels of depression and slightly lower for infants born to fathers with diabetes and greater levels of fast food consumption [25]. The goal of this study is to see if there’s a link between maternal and paternal diabetes, maternal hypertension, maternal alcohol use, mother depression, and paternal fast food intake and newborn birth outcomes. Preconception health promotion activities can target these characteristics in order to enhance birth outcomes, which will benefit the health of future generations.

Maternal nutritional deficiencies, particularly iron and folates, are common in LMICs. Anemia in women from LMICs is due to low dietary intake of bioavailable iron combined with endemic infectious diseases such as helminthiasis, which puts women at increased risk during pregnancy. Low preconception hemoglobin and ferritin levels increase the risk of poor fetal growth and low birth weight [26]. Similarly, folate deficiency can lead to the development of neural tube defects in the fetus. Other micronutrients such as zinc, vitamin B, and calcium have been found to improve maternal and newborn outcomes when supplementation is provided during pregnancy; however, their impact during the preconception period has not been established [27]. The findings of this study will support the idea of increasing women’s preconception nutritional status by delivering critical nutritional supplements throughout the preconception period, which can assist women to start their pregnancy in the best possible health.

Improved reproductive health and planning is the fundamental component of preconception care, and starting early interventions in the preconception period may improve the participants’ knowledge and self-efficacy toward the need for better health before and during pregnancy, which may contribute to those favorable outcomes. Although policies and guidelines on preconception care are available, this study intends to implement the recommendations and good clinical practice guidelines in a low-resource rural community setting of India. So this study will fill the gap in the continuum of care, particularly for women who are not pregnant. Evidence also indicates that prenatal care is frequently too late to prevent negative health consequences for developing fetuses. The goal of the study is to introduce nutrition and other lifestyle interventions during the preconception period, which will be the best time to promote maternal health and ensure a healthy pregnancy. This intervention is cost-effective but at the same time will be very challenging to implement before pregnancy in India’s low-resource setting.

### Limitations

The study will be a nonrandomized clinical trial which may limit the validity of the study outcome, and the study setting will be limited to one district of the Odisha state, India. The study needs a long duration of a minimum of 2 years to measure the effects on birth outcome; hence, there is more possibility of nonadherence to the preconception services as well as noncompliance for routine antenatal care. However, those cases will be followed up by the local community nurse-midwives and research team members, and necessary counseling sessions will be conducted for adherence to care. Additionally, preconception care needs tremendous effort and cooperation from the field health care women and their partners. Thus, exploring facilitators and barriers to the implementation of the preconception care intervention is a vital step of this proposed project.

### Future Plans

As an extension of the outcome of this study, training can be provided to concerned community health workers who will provide extensive support to the women using this preconception care intervention for better health outcomes, mostly in a low-resource community setting. Also, the development of age-appropriate educational modules for use in school health education programs and integration of reproductive health messages into existing health promotion campaigns for targeted adolescents is a long-term goal.

### Conclusions

The eligible couples will adopt strategies to improve their preconception health through preconception care and counseling. Structured preconception care in community settings has the potential to prevent unfavorable pregnancy and childbirth consequences. Finally, maternal and paternal health are likely to have a significant impact on maternal nutrition and birth outcomes.

## Abbreviations

LMICs: low- and middle-income countries
MHC: maternal health care
